# No Association Between Allergic Diseases and Constipation in Japanese Ulcerative Colitis Patients: A Cross-Sectional Study

**DOI:** 10.7759/cureus.55912

**Published:** 2024-03-10

**Authors:** Sen Yagi, Shinya Furukawa, Seiyuu Suzuki, Katsuhisa Ohashi, Hideomi Tomida, Yasunori Yamamoto, Eiji Takeshita, Yoshio Ikeda, Yoichi Hiasa

**Affiliations:** 1 Internal Medicine, Saiseikai Imabari Hospital, Imabari, JPN; 2 Health Services Center, Ehime University, Matsuyama, JPN; 3 Gastroenterology, Sumitomo Besshi Hospital, Niihama, JPN; 4 Surgical Gastroenterology, Ohashi Clinic, Niihama, JPN; 5 Endoscopy Center, Ehime University Hospital, Toon, JPN; 6 Gastroenterology and Metabology, Ehime University Graduate School of Medicine, Toon, JPN

**Keywords:** ulcerative colitis, allergy, inflammatory bowel disease, constipation, allergic diseases

## Abstract

Background: Constipation is a common gastrointestinal symptom in patients with ulcerative colitis (UC). Several studies on the general population have demonstrated a link between allergic diseases and constipation. However, evidence regarding the association between allergic diseases and constipation in UC is limited. This study aims to evaluate this issue in Japanese patients with UC.

Methods: This cross-sectional study recruited consecutive 387 patients with UC. We used a self‐administered questionnaire to estimate the prevalence of physician-diagnosed allergic diseases. The definition of constipation was based on Rome I criteria and/or medication for constipation.

Results: The prevalence of constipation was 12.5%. The prevalence rates of asthma, atopic dermatitis, pollen allergy, food allergy, and drug allergy were 11.8%, 9.0%, 36.3%, 6.2%, and 8.3%, respectively. Allergic diseases were not associated with constipation (adjusted odds ratio [OR] with asthma (adjusted OR 0.98 [95% confidence interval [CI] 0.27-2.80]), atopic dermatitis (adjusted OR 0.67 [95% CI 0.10-2.56]), pollen allergy (adjusted OR 0.92 [95% CI 0.41-1.97]), food allergy (adjusted OR 0.76 [95% CI 0.11-2.95]), and drug allergy (adjusted OR 1.06 [95% CI 0.28-3.24]). Additionally, the number of allergic diseases was not associated with the prevalence of constipation.

Conclusions: In Japanese UC patients, no association between allergic diseases and constipation was found.

## Introduction

Allergic diseases, such as asthma, atopic dermatitis, pollen allergy, food allergy, and drug allergy, are systemic diseases caused by an impaired immune system. The incidence of these diseases continues to increase dramatically around the world [1], resulting in considerable burden to society [2]. Constipation is a common gastrointestinal condition in daily clinical practice that has been associated with chronic kidney disease [3], incidence of cardiovascular diseases [4], and all-cause mortality [5]. Allergic diseases and constipation are both significant public health concerns.

Several studies have reported a positive association between allergic diseases and constipation in childhood [6]. The prevalence of food allergy was 15% in subjects with constipation [7]. Constipated individuals are at higher risk for asthma, atopic dermatitis, and allergic rhinitis [8-11]. Nevertheless, the findings regarding the association between allergic diseases and constipation are limited and inconsistent in adult studies.

Four studies have reported that the prevalence of allergic symptoms is higher in patients with ulcerative colitis (UC) compared to healthy controls [12-14]. The number of patients with ulcerative colitis is increasing [15]. Although high stool frequency is a common gastrointestinal symptom of UC, previous study have shown that roughly one third to one half of patients have constipation symptoms [16]. However, the physiology of constipation in patients with UC is poorly understood [17]. Abnormalities of the gut microbiota are associated with UC, constipation and allergic diseases, respectively. Constipation alters the gut microbiota and increases susceptibility to immune abnormalities [18], while subclinical inflammation is associated with constipation [19]. It is therefore clinically important to investigate the association between allergic disease and constipation in UC. Thus, we hypothesize that the prevalence of allergic diseases might affect constipation in patients with UC. However, no survey regarding the linkage between allergic diseases and constipation in UC patients exists.

The aim of this study is to investigate this issue in Japanese UC patients.

## Materials and methods

Study population

This survey was a cross-sectional analysis using baseline dataset from a prospective cohort study conducted from 2015 to 2019. A total of 387 UC patients who visited Ehime University Hospital and several affiliated hospitals and clinics in Ehime prefecture were enrolled in the cohort study. Inpatients and outpatients who were diagnosed with UC based on mainly endoscopic findings and considered capable of answering a self-administered questionnaire were included. Written informed consent was obtained by well-trained staff from all patients. The study protocol was developed in accordance with the 1964 Declaration of Helsinki, and was approved by the Ethics Committee of Ehime University Hospital (approval number 1505011). This study is also registered in the University Hospital medical information network (UMIN 000051334).

Data source

Ninety-eight patients declined to participate in the colonoscopy and blood tests and were unable to complete the questionnaire, leaving a total of 289 participants (167 males and 122 females). A certified endoscopist evaluated the mucosal status by total colonoscopy. Mucosal healing (MH) was defined as a Mayo Endoscopic Subscore (MES) of 0 [20]. Using a self-administered questionnaire, we assessed age, sex, body mass index (BMI), current smoking status, and current drinking habits. Information on disease extent, duration, medication for UC (including 5-aminosalicylates, prednisolone, thiopurines, and tumor necrosis factor (TNF)-α monoclonal antibody), and clinical remission (CR) was obtained from medical records.

Definition of allergic disease

To obtain information regarding allergic diseases, this study used a self‐administered questionnaire including the following questions: “Have you ever been diagnosed with asthma, atopic dermatitis, pollen allergy, food allergy, or drug allergy by your doctor?”

Definition of constipation

The diagnosis of constipation was based on criteria established by expert consensus called the Rome I criteria. In addition, the self-administered questionnaire was used to confirm the presence or absence of medication for constipation.

Statistical analysis

The crude odds ratio (OR) and their 95% confidence interval (CI) for constipation in relation to each allergic disease and the number of allergic diseases were analyzed using logistic regression analysis. Multiple logistic regression analysis was used to adjust for potential confounders. Age, sex, BMI, current smoking status, and current drinking habits were selected as potential confounders. Patients were divided into three groups based on the number of allergies, 0 (reference), 1, and 2 or more. Two-sided p < 0.05 was considered statistically significant in all analyses. All statistical analyses were performed using the SAS software package ver. 9.4 (SAS Institute, Cary, NC, USA).

## Results

Table 1 presents the characteristics of the 289 study participants. The percentage of men was 57.8% (167/289) in this cohort. The mean age and BMI were 50.1 years and 22.63, respectively. The prevalence rates of constipation, complete MH (MES 0), and CR were 12.5% (36/289), 24.6% (71/289), and 59.5% (172/289), respectively. The prevalence rates of asthma, atopic dermatitis, pollen allergy, food allergy, and drug allergy were 11.8% (34/289), 9.0% (26/289), 36.3% (105/289), 6.2% (18/289), and 8.3% (24/289), respectively. 

**Table 1:** Clinical characteristics of 289 study participants BMI, body mass index; UC, ulcerative colitis; SD, standard deviation; TNF, tumor necrosis factor; Other: right-sided, segmental colitis, and postoperative patients (lack of any preoperative medical records for postoperative patients)

Variables	Mean ± SD or n (%)
Age (years)	50.1 ± 16.0
Male (%)	167 (57.8)
Disease extent (n; pancolitis/left-sided/proctitis/other)	120/78/84/7
Duration of UC (years)	8.3 ± 8.4
BMI	22.63 ± 4.60
Current smoking	21 (7.3)
Current drinking	118 (40.8)
Medication for UC	
5-Aminosalicylates	264 (91.4)
Prednisolone	58 (20.1)
Thiopurines	42 (14.5)
TNF-α monoclonal antibody	16 (5.5)
Mayo Endoscopic Subscore (MES)	1.22 ± 0.91
Mucosal healing (MES 0)	71 (24.6)
Clinical remission	172 (59.5)
Constipation (%)	36 (12.5)
Medication for constipation (%)	30 (10.4)
Allergic diseases	
Asthma (%)	34 (11.8)
Atopic dermatitis (%)	26 (9.0)
Pollen allergy (%)	105 (36.3)
Food allergy (%)	18 (6.2)
Drug allergy (%)	24 (8.3)
Number of allergy diseases	1.7 ± 0.8

Table 2 presents the crude and adjusted ORs and 95% CIs for allergic diseases related to constipation. The prevalence rates of constipation among asthma, atopic dermatitis, pollen allergy, food allergy, and drug allergy were 11.8% (4/34), 7.7% (2/26), 11.4% (12/105), 11.1% (2/18), and 16.7% (4/24), respectively. Allergic disease groups were not associated with constipation: asthma (adjusted OR 0.98, 95% CI 0.27-2.80), atopic dermatitis (adjusted OR 0.67, 95% CI 0.10-2.56), pollen allergy (adjusted OR 0.92, 95% CI 0.41-1.97), food allergy (adjusted OR 0.76, 95% CI 0.11-2.95), and drug allergy (adjusted OR 1.06, 95% CI 0.28-3.24). Smoking status was not associated with constipation. After exclusion for current smokers, similarly, no association between allergic diseases and constipation was found.

**Table 2:** Crude and adjusted odds ratios and 95% confidence intervals for allergy diseases related to constipation Adjusted for age, sex, body mass index, current drinking, current smoking, and use of steroid. OR, odds ratio; CI, confidence interval

Variable	Prevalence (%)	Crude OR (95% CI)	Adjusted OR (95% CI)
Constipation			
Asthma, n (%)			
No	32/255 (12.6)	1.00	1.00
Yes	4/34 (11.8)	0.92 (0.26–2.55)	0.98 (0.27–2.80)
Atopic dermatitis, n (%)			
No	34/263 (12.9)	1.00	1,00
Yes	2/26 (7.7)	0.56 (0.09–2.01)	0.67 (0.10–2.56)
Pollen allergy, n (%)			
No	24/184 (13.0)	1.00	1.00
Yes	12/105 (11.4)	0.86 (0.40–1.77)	0.92 (0.41–1.97)
Food allergy, n (%)			
No	34/271 (12.6)	1.00	1.00
Yes	2/18 (11.1)	0.87 (0.13–3.25)	0.76 (0.11–2.95)
Drug allergy, n (%)			
No	32/265 (12.1)	1.00	1.00
Yes	4/24 (16.7)	1.45 (0.41–4.15)	1.06 (0.28–3.24)

Table 3 presents the crude and adjusted ORs and 95% CIs for number of allergic diseases in relation to constipation. The prevalence rates of constipation among number of allergic diseases, zero, one, and two or more were 11.6% (17/146), 14.9% (14/94), and 10.2% (5/49), respectively. The number of allergic diseases was not associated with constipation (adjusted OR with number of allergic diseases, one: adjusted OR 1.31, 95% CI 0.59-2.87), and two or more (adjusted OR 0.84, 95% CI 0.25-2.41).

**Table 3:** Crude and adjusted odds ratios and 95% confidence intervals for number of allergy diseases in relation to constipation Adjusted for age, sex, body mass index, current drinking, current smoking, and use of steroid. OR, odds ratio; CI, confidence interval

Variable	Prevalence (%)	Crude OR (95% CI)	Adjusted OR (95% CI)
Number of allergy diseases			
Constipation			
0	17/146 (11.6)	1.00	1.00
1	14/94 (14.9)	1.33 (0.61–2.84)	1.31 (0.59–2.87)
2 or more	5/49 (10.2)	0.86 (0.27–2.33)	0.84 (0.25–2.41)
p for trend			0.98

## Discussion

In this study, no association was found between allergic diseases and constipation in patients with UC. Although there have been reports on the relationship between allergic diseases and constipation in healthy childhood subjects, this is the first study to investigate this issue in patients with UC.

The prevalence of allergic diseases has been increasing considerably, with allergic rhinitis being the most common allergic disease in the United States (US) and other industrialized countries [21]. Asthma is one of the most common chronic diseases in childhood [22]. The prevalence of food allergy is 6.7% in the US [23]. Up to 1% of the population in the US and Europe experiences chronic urticaria during their lifetime [24].

The prevalence of allergic disease in inflammatory bowel disease (IBD) varies widely across studies. The prevalence of allergic symptoms was higher in the patient group (56%) compared to the control group (18%) [13]. In a study of 577 patients with IBD, 30 had asthma, five had allergic rhinitis and 386 had other allergies (other allergies such as food, drugs, pollen, latex) [25]. Additionally, the prevalence of constipation was lower than previous study. In our study, the prevalence of allergic diseases and constipation tended to be lower than in previous studies with IBD. The definitions of allergic diseases and constipation, and medication for UC may have resulted in their respective lower prevalence rates.

Particularly in children, an increasing number of reports have suggested a relationship between allergic diseases and constipation [6]. In one report, 15% of patients with constipation had a diagnosed food allergy [7]. In addition, patients with constipation had a 36.2-fold higher risk of asthma than patients without constipation [8].

The association between allergic diseases and constipation in adults is limited and inconsistent. Three Taiwanese cohort studies reported that constipation was a risk factor for allergic dermatitis [10], allergic rhinitis [11], and asthma [9]. On the other hand, in a US study, food allergies including peanut, eggs, and allergies to dogs and cockroaches are inversely associated with the prevalence of constipation [26].

Several allergic diseases, primarily asthma and atopic dermatitis, have been associated with IBD, including UC [27-30]. Additionally, a Korean study has shown that the number of allergic diseases is correlated with the incidence of UC [31]. In a cross-sectional study of 125 patients with UC, the prevalence of constipation was 46% [16]. However, no study has investigated the association between allergic diseases and constipation in patients with UC, despite the fact that both constipation and allergic diseases are linked to UC.

Several hypotheses have been proposed regarding the association between allergic disease and constipation, including the influence of the intestinal microbiota [32], damage to the mucosal barrier [33], immune system regulation [34], and diet and nutrition; however, no definitive mechanism has been elucidated yet. As this study employed a hospital-based cohort, medication for UC, allergic diseases, and constipation might mask the true association between allergic diseases and constipation.

This study has several limitations. First, this was a cross-sectional analysis and we therefore cannot infer a causal relationship between allergic diseases and constipation. Second, the sample size is small for this statistic, which might have caused a type II error. Third, the information regarding allergic diseases and medication for constipation was obtained using a self-administered questionnaire, which may have led to misclassification. Additionally, we used ROME I criteria to assess the prevalence of constipation in this study. Fourth, as this cohort included longstanding UC, medication for UC and other comorbidities might have affected both allergic diseases and constipation. Fifth, we could not perform skin prick tests to diagnose the allergic diseases. Finally, there are variations in the prevalence and definition of constipation, and these patients were selected by questionnaire.

## Conclusions

In Japanese patients with UC, there was no association between each allergic disease and constipation. Furthermore, the number of allergic diseases was not associated with the prevalence of constipation. Since this cohort consisted of patients with UC who were already being treated, it is possible that medications such as those used to treat UC may have masked the true association between allergic disease and constipation. Further investigation on this issue is warranted.
